# A Review on Various Topics on the Thermal Grill Illusion

**DOI:** 10.3390/jcm10163597

**Published:** 2021-08-16

**Authors:** Dong Ah Shin, Min Cheol Chang

**Affiliations:** 1 Department of Neurosurgery, College of Medicine, Yonsei University, Seodaemun-gu, Seoul 03722, Korea; CISTERN@yuhs.ac; 2 Department of Physical Medicine and Rehabilitation, College of Medicine, Yeungnam University, Namku, Taegu 42415, Korea

**Keywords:** thermal grill illusion, pain, temperature, review

## Abstract

The thermal grill illusion (TGI) is a paradoxical perception of burning heat and pain resulting from the simultaneous application of interlaced warm and cold stimuli to the skin. The TGI is considered a type of chronic centralized pain and has been used to apply nociceptive stimuli without inflicting harm to human participants in the study of pain mechanisms. In addition, the TGI is an interesting phenomenon for researchers, and various topics related to the TGI have been investigated in several studies, which we will review here. According to previous studies, the TGI is generated by supraspinal interactions. To evoke the TGI, cold and warm cutaneous stimuli should be applied within the same dermatome or across dermatomes corresponding to adjacent spinal segments, and a significant difference between cold and warm temperatures is necessary. In addition, due the presence of chronic pain, genetic factors, and sexual differences, the intensity of the TGI can differ. In addition, cold noxious stimulation, topical capsaicin, analgesics, self-touch, and the presence of psychological diseases can decrease the intensity of the TGI. Because the TGI corresponds to chronic centralized pain, we believe that the findings of previous studies can be applied to future studies to identify chronic pain mechanisms and clinical practice for pain management.

## 1. Introduction

The thermal grill illusion (TGI), first introduced in 1896, is the perception of burning heat and pain that arises from the simultaneous cutaneous application of a grill with interlaced warm (38–42 °C) and cool (18–22 °C) bars [1,2,3]. Although cold and warm cutaneous stimulations are felt as coolness and warmth in isolation, respectively, their spatial combination (when placing a hand on the interlaced grill) often creates a thermo-nociceptive prickling sensation (burning heat and pain) [1,2,3].

Considering that the temperatures of the interlaced warm and cool bars are innocuous, peripheral nociceptors are unlikely to be activated. Therefore, pain due to the TGI is believed to be a purely central phenomenon [4]. Therefore, it was suggested that TGI-related pain would be useful for evaluating centrally sensitized pain. In addition, the TGI has been used to apply nociceptive stimuli without inflicting harm to human participants in studies investigating pain mechanisms [5,6]. Centralized pain refers to changes within the central nervous system that amplify the peripheral input or generate pain perception in the absence of a noxious stimulation and is one of the mechanisms underlying the development and maintenance of chronic pain [7]. Therefore, the TGI is considered a type of chronic centralized pain. As mentioned above, the TGI is an interesting phenomenon for researchers; thus, to uncover the curiosity of this phenomenon, various topics related to the TGI have been investigated in several studies. A review of these previous studies would be meaningful because the findings of previous studies could be used to investigate the characteristics or mechanisms of chronic pain in future studies and applied to real clinical practice for managing chronic pain. Therefore, in the current study, we reviewed previous studies on the TGI.

## 2. Methods

PubMed was searched for previous studies from 1 January 1980 to 9 August 2021, using the term “Thermal grill illusion.” We reviewed human studies in which TGI-related phenomena were investigated and the TGI was used to conduct research on pain.

## 3. Results

### 3.1. The Underlining Mechanisms of the TGI

Two major theories on the development of the TGI have been suggested: the “addition or convergence theory” and “disinhibition or unmasking theory” [8,9,10,11,12].

The addition or convergence theory suggests that the TGI is produced by a polymodal pathway that linearly summates warm and cold sensory inputs [8,12]. The strength of synthetic heat perception increases depending on the absolute difference between cold and warm temperatures [8,12]. Both warm and cold cutaneous simulations activate the same polymodal pathway, and these individual stimulations are summated [8,12]. A more intense stimulation by summation can be perceived as painful. The larger the difference between the warm and cold temperatures, the stronger the TGI evoked [8,12]. Green et al. revealed that the intensity of the TGI is similar to the sum of the perceived intensities of the warm and cool component temperatures [12].

Disinhibition or unmasking theory involves two serial steps [9,10]. First, warm stimulation inhibits the response of neurons to cold temperatures in the spinal cord, which subsequently prevents signaling to the ventromedial posterior nucleus of the thalamus by inhibiting ventral caudal subdivision of the mediodorsal nucleus (MDvc) [9,10]. Second, uncontrolled excessive stimuli from the MDvc to the anterior cingulate cortex and insula lead to TGI-induced pain [9,10]. Craig et al. found that when both 20 and 40 °C stimuli were simultaneously applied to anesthetized cats, the activity of neurons responding to cold temperatures in the spinal cord was reduced by 50% [9].

### 3.2. The Area in which the Perception of the TGI Originates

Ferrè et al. evaluated whether the perception of the TGI originates at the spinal or supraspinal levels. The inhibition of nociceptive input by concomitant non-nociceptive somatosensory input is known to have a strong spinal component [13]. For this reason, they proposed that if the afferent input underlying the TGI originates at the spinal level, then the TGI is inhibited by a concomitant non-nociceptive somatosensory input (tactile stimulation). By contrast, if the TGI is provoked by the supraspinal process, a concomitant tactile stimulation would not affect the TGI. To remove the effect of touch on the TGI, instead of the conventional TGI based on mechanical contact between the skin and hot and cold bars, the authors applied a novel radiant thermal stimulation. In their study, TGI was not influenced by the presence of tactile stimulation. They concluded that TGI-induced pain would result from supraspinal interactions. However, because Ferrè et al. did not demonstrate that tactile stimulation applied in their study could inhibit nociceptive input, the conclusion might not be robust.

### 3.3. Changes in the TGI Following Segmental Distance of Cold and Warm Afferents

Fardo et al. evaluated the change in the TGI response according to the segmental distance between cold and warm afferents [14]. They found that when cold and warm cutaneous stimuli were delivered within the same dermatome or across dermatomes corresponding to adjacent spinal segments, the TGI was manifested. By contrast, no TGI effect was observed when cold and warm stimuli were projected from the nonadjacent spinal segments. These results suggest that the degree of the TGI is modulated by the segmental distance between cold and warm afferents, and thermal-nociceptive interactions appear to occur depending on low-level convergence mechanisms acting within a single or neighboring spinal segment. However, Defrin et al. used a similar method to that used in the study by Fardo et al. but found the opposite result [3]. Defrin et al. applied cold and warm stimulation in either the proximal or distal upper extremities separately [3]. In their study, the TGI response was manifested, and they insisted that even cold and warm stimuli were projected from distinct spinal segments, evoking the TGI. However, although the direct distance between the proximal and distal portions of the upper extremity is long, there is not much difference in the spinal segmental differences (proximal portion: C5, distal: C5–C8) [15]. Therefore, we believe that Defrin et al. did not perform their study using an appropriate method. Cold and warm stimuli should have been applied to dermatomes related to different spinal segments based on the accurate knowledge of dermatomes.

### 3.4. The Response to the TGI in Patients with Chronic Pain

Two studies evaluated whether chronic pain affected the response to the TGI, with conflicting results. Sumracki et al. evaluated the difference in the response to the TGI between patients with chronic pain (*n* = 18) and pain-free participants (*n* = 16) [16]. They compared the responses to the TGI in patients with chronic pain with those in pain-free participants. Although there was a minimal difference, the intensity of TGI-related pain and degree of discomfort were significantly lower in patients with chronic pain than in pain-free participants. Sumracki et al. suggested that patients with chronic pain have an altered central integration of ascending pain signals. This proposal is correlated with long-term functional and structural changes in the central nervous system. In contrast to the results of this study, the case report by Heavner et al. showed the opposite response [17]. They reported that a patient with chronic pain due to complex regional pain syndrome experienced an intolerable burning sensation on the thermal grill; however, this result might be unreliable because this study involved only one patient with chronic pain. More studies are needed to clarify whether chronic pain increases the intensity of the pain response to the thermal grill or decreases it. Furthermore, the response to the TGI following pain duration would be an interesting topic for future studies.

### 3.5. Modulation of TGI-Induced Pain

In previous studies on TGI-related topics, the shape and quantity of the alternating warm and cold bars varied extensively and were not important factors for evoking the TGI [18]. However, the spinal segmental difference between warm and cold temperatures and the spatial pattern of thermal stimulation are crucial factors in the generation of the TGI [14,19].

Harper et al. evaluated the effect of conditioned pain modulation on TGI [20]. Before placing 18 healthy participants’ right volar forearm on a thermal grill, comprising innocuous alternating warm (42 °C) and cool (18 °C) bars, the left hand was placed in noxious cold water (6 °C). When the left hand was placed in a neutral water bath (33 °C), cold noxious stimulation with cold water significantly reduced pain and discomfort by 47.0% and 56.6%, respectively. Descending inhibitory pathways appear to be activated during conditioned pain modulation (cold noxious cutaneous stimulation), which suppresses the ascending innocuous thermal information. Additionally, in the studies by Harper et al., most reported sensations by a thermal grill were hot, burning, stinging, sharp, and aching.

Schaldemose et al. recruited 80 healthy participants and topically applied capsaicin and ethanol (control) on the volar forearms in a randomized order [21]. Capsaicin induced hyperalgesia, allodynia, and decreased cold and cold pain sensations. They placed the volar forearms on a thermal grill (10/40 °C). On the control side, the thermal grill induced more pain and burning sensations, although after capsaicin application, the thermal grill induced less pain and burning sensations. Capsaicin reduces the activity of the C-nociceptor; consequently, the sensation of temperature transferred via C fibers decreases. The reduced C-fiber activity by topical capsaicin decreased the transfer of thermal information to the spine and brain. Consequently, the thermal-nociceptive interaction, which is necessary to evoke the TGI, is decreased. Additionally, Kern et al. demonstrated that analgesics modulate TGI [5]. They revealed that the intravenous administration of morphine (0.1 mg/kg) induced the reduction of TGI-induced pain, which was correlated with the reduction of cold pain.

Harper et al. [22] reported that coolness involves both the production and protection of TGI-induced pain. Adaptation to the grill’s cool bars significantly reduced TGI-induced pain, which indicates that coolness underlies the development of TGI-induced pain. On the other hand, the feeling of coolness during the illusion was significantly associated with less TGI-induced pain.

In addition, Kammers et al. reported a dramatic 64% reduction in TGI-induced pain in the right hand on a thermal grill at self-touch with the right and left hands [23]. In addition, the temperature difference between the cold and warm bars induced different intensities of TGI-induced pain [24]. Leung et al. reported that the degree of pain was significantly higher in 20/40 and 18/42 °C thermal grills than in 22/38 and 24/36 °C thermal grills [24].

As described above, various factors, such as noxious cold temperature, capsaicin, analgesic, touch sensation, coolness, and temperature of the thermal grill, can modulate the intensity of TGI-induced pain [5,20,21,22,23,24].

### 3.6. The Response to the TGI in Patients with Psychological Disorders

Bekrater-Bodmann et al. reported alterations in the TGI due to borderline personality disorder (BPD) [25]. They recruited 29 patients with current BPD, 19 patients with BPD in remission, and 22 healthy participants. They found that the intensities of TGI-induced pain and unpleasantness were lower in patients with current BPD than in those with BPD in remission and healthy participants. However, the general ability to perceive TGI in patients with current BPD was not affected. The intensity of the TGI was negatively correlated with dissociation and traumatization in patients with current BPD. In addition, Boettger et al. compared the TGI response between 18 patients with acute paranoid schizophrenia and 18 matched controls [26]. Compared with controls, temperature differentials for the perception of the TGI and cold and heat pain thresholds were increased in patients with schizophrenia. In another study, Boettger et al. recruited 18 patients with major depressive disorder (MDD) and 18 matched controls and evaluated the pain threshold and intensity of TGI-induced pain [27]. In patients with MDD, cold and heat pain thresholds were significantly increased compared to those in controls. In addition, patients with MDD experienced less TGI pain despite increased stimulus intensity. In contrast to these results, in another study by Boettger et al. and Pinerua-Shuhaibar et al., transiently mildly depressed people or people in a sad mood state showed an increase in TGI-induced pain [28,29].

### 3.7. Sexual Difference in the TGI

Several previous studies have shown that women are more sensitive to painful stimuli than men across both patient groups and healthy participants [30,31,32]. Furthermore, Girard-Tremblay et al. showed differences in brain activation in response to painful stimuli. They reported that men tend to activate prefrontal cortex-mediated threat-control circuits, but in women, emotion-processing centers are activated to cope with pain unpleasantness [33]. This result indicates that sexual differences in psychological mechanisms may affect responses to pain sensitivity and endurance.

Sexual differences in TGI were investigated by Averbeck et al. [34]. They recruited 78 female and 58 male undergraduate students. The TGI was evoked by placing the palm of the right hand on a thermal grill (20/40 °C). TGI-induced pain was more pronounced in women than in men, and the thermal pain threshold was lower in women than in men.

### 3.8. Genetic Factors Contributing to the Intensity of TGI-Induced Pain

Several studies have shown that various genes play important roles in determining pain sensitivity and susceptibility to the development of chronic pain [35,36,37,38]. It has been reported that specific genetic polymorphisms involving serotonin and dopamine receptors and serotonin transporters are more frequently observed in patients with fibromyalgia [35,36].

To date, only one study has reported a genetic effect on TGI-induced pain. Lindstedt et al. evaluated the relationship between the level of expression of the 5-HT transporter (5-HTT) and perception of the TGI. Forty-four healthy participants (21 participants: low 5-HTT-expressing group; 23 participants: high 5-HTT-expressing group) were recruited [39]. Females with low 5-HTT expression perceived TGI as significantly less unpleasant than females with high 5-HTT expression.

## 4. Discussion

We reviewed mechanisms and phenomena related to the TGI. In pain research, one of the major limitations of pain models is that they activate peripheral nociceptive inputs. Therefore, it is difficult to separate peripheral and central processes of pain. Using TGI helps apply centralized pain without any stimulating peripheral pain input or processing. Results of previous TGI studies are consistent with those of other studies in that the chronic pain threshold can differ according to the presence of chronic pain and psychological disorders, sex, and genetic expression [40,41,42]. We believe that the TGI can be useful for supporting or confirming previous results related to chronic pain. In addition, the TGI would be helpful for evaluating the effect of pharmacological treatments or physical modalities on reducing chronic centralized pain. Furthermore, it might be useful for evaluating the presence of psychological factors that affect the development of chronic pain. Finally, it can be used to identify genetic factors affecting the sensitivity and susceptibility to the development of chronic pain. However, studies using the TGI are limited in that measuring the pain degree and character when applying TGI is subjective, depending on participants’ reports.

We reviewed several previous studies on the TGI. In summary, the TGI perception is generated by supraspinal interactions. In addition, the presence of chronic pain, genetic factors, and sexual differences can affect the intensity of the TGI. In addition, cold noxious stimulation, topical capsaicin, analgesics, self-touch, and the presence of psychological diseases can lower the degree of TGI. In addition, to evoke the TGI, cold and warm cutaneous stimuli should be applied within the same dermatome or across dermatomes corresponding to adjacent spinal segments, and a significant difference between cold and warm temperatures is necessary. Considering that the TGI corresponds to chronic centralized pain, we believe that some findings in previous studies can be used to investigate chronic pain mechanisms in future studies and to adjust to real clinical practice for pain management.

## Data Availability

No new data were created or analyzed in this study. Data sharing is not applicable to this article.

## References

[1] Alrutz S. (1898). On the temperature-senses. Mind.

[2] Bach P., Becker S., Kleinböhl D., Hölzl R. (2011). The thermal grill illusion and what is painful about it. Neurosci. Lett..

[3] Defrin R., Benstein-Sheraizin A., Bezalel A., Mantzur O., Arendt-Nielsen L. (2008). The spatial characteristics of the painful thermal grill illusion. Pain.

[4] Kern D., Pelle-Lancien E., Luce V., Bouhassira D. (2008). Pharmacological dissection of the paradoxical pain induced by a thermal grill. Pain.

[5] Kern D., Plantevin F., Bouhassira D. (2008). Effects of morphine on the experimental illusion of pain produced by a thermal grill. Pain.

[6] Kong Y., Posada-Quintero H.F., Chon K.H. Pain Detection using a Smartphone in Real Time. Proceedings of the 2020 42nd Annual International Conference of the IEEE Engineering in Medicine & Biology Society (EMBC).

[7] Eller-Smith O.C., Nicol A.L., Christianson J.A. (2018). Potential Mechanisms Therapeutic Interventions. Front. Cell Neurosci..

[8] Bouhassira D., Kern D., Rouaud J., Pelle-Lancien E., Morain F. (2005). Investigation of the paradoxical painful sensation (‘illusion of pain’) produced by a thermal grill. Pain.

[9] Craig A.D., Bushnell M.C. (1994). The thermal grill illusion: Unmasking the burn of cold pain. Science.

[10] Craig A.D., Reiman E.M., Evans A., Bushnell M.C. (1996). Functional imaging of an illusion of pain. Nature.

[11] Fardo F., Beck B., Allen M., Finnerup N.B. (2020). Beyond labeled lines: A population coding account of the thermal grill illusion. Neurosci. Biobehav. Rev..

[12] Green B.G. (2002). Synthetic heat at mild temperatures. Somatosens. Mot. Res..

[13] Ferrè E.R., Iannetti G.D., van Dijk J.A., Haggard P. (2018). Ineffectiveness of tactile gating shows cortical basis of nociceptive signaling in the Thermal Grill Illusion. Sci. Rep..

[14] Fardo F., Finnerup N.B., Haggard P. (2018). Organization of the Thermal Grill Illusion by Spinal Segments. Ann. Neurol..

[15] Whitman P.A., Adigun O.O. (2021). Anatomy, Skin, Dermatomes. StatPearls.

[16] Sumracki N.M., Buisman-Pijlman F.T., Hutchinson M.R., Gentgall M., Rolan P. (2014). Reduced response to the thermal grill illusion in chronic pain patients. Pain Med..

[17] Heavner J.E., Calvillo O., Racz G.B. (1997). Thermal grill illusion and complex regional pain syndrome type I (reflex sympathetic dystrophy). Reg. Anesth..

[18] Li X., Petrini L., Wang L., Defrin R., Arendt-Nielsen L. (2009). The importance of stimulus parameters for the experience of the thermal grill illusion. Neurophysiol. Clin..

[19] Marotta A., Ferrè E.R., Haggard P. (2015). Transforming the thermal grill effect by crossing the fingers. Curr. Biol..

[20] Harper D.E., Hollins M. (2017). Conditioned pain modulation dampens the thermal grill illusion. Eur. J. Pain.

[21] Schaldemose E.L., Horjales-Araujo E., Svensson P., Finnerup N.B. (2015). Altered thermal grill response and paradoxical heat sensations after topical capsaicin application. Pain.

[22] Harper D.E., Hollins M. (2014). Coolness both underlies and protects against the painfulness of the thermal grill illusion. Pain.

[23] Kammers M.P., de Vignemont F., Haggard P. (2010). Cooling the thermal grill illusion through self-touch. Curr. Biol..

[24] Leung A.Y., Wallace M.S., Schulteis G., Yaksh T.L. (2005). Qualitative and quantitative characterization of the thermal grill. Pain.

[25] Bekrater-Bodmann R., Chung B.Y., Richter I., Wicking M., Foell J., Mancke F., Schmahl C., Flor H. (2015). Deficits in pain perception in borderline personality disorder: Results from the thermal grill illusion. Pain.

[26] Boettger M.K., Grossmann D., Bär K.J. (2013). Increased cold and heat pain thresholds influence the thermal grill illusion in schizophrenia. Eur. J. Pain.

[27] Boettger M.K., Grossmann D., Bär K.J. (2013). Thresholds and perception of cold pain, heat pain, and the thermal grill illusion in patients with major depressive disorder. Psychosom. Med..

[28] Boettger M.K., Schwier C., Bär K.J. (2011). Sad mood increases pain sensitivity upon thermal grill illusion stimulation: Implications for central pain processing. Pain.

[29] Piñerua-Shuhaibar L., Villalobos N., Delgado N., Rubio M.A., Suarez-Roca H. (2011). Enhanced central thermal nociception in mildly depressed nonpatients and transiently sad healthy subjects. J. Pain.

[30] Mogil J.S. (2012). Sex differences in pain and pain inhibition: Multiple explanations of a controversial phenomenon. Nat. Rev. Neurosci..

[31] Racine M., Tousignant-Laflamme Y., Kloda L.A., Dion D., Dupuis G., Choinière M. (2012). A systematic literature review of 10 years of research on sex/gender and experimental pain perception-part 1: Are there really differences between women and men?. Pain.

[32] Riley J.L., Gilbert G.H., Heft M.W. (1998). Orofacial pain symptom prevalence: Selective sex differences in the elderly?. Pain.

[33] Girard-Tremblay L., Auclair V., Daigle K., Léonard G., Whittingstall K., Goffaux P. (2014). Sex differences in the neural representation of pain unpleasantness. J. Pain.

[34] Averbeck B., Seitz L., Kolb F.P., Kutz D.F. (2017). Sex differences in thermal detection and thermal pain threshold and the thermal grill illusion: A psychophysical study in young volunteers. Biol. Sex Differ..

[35] Buskila D. (2007). Genetics of chronic pain states. Best Pract. Res. Clin. Rheumatol..

[36] Matsuda J.B., Barbosa F.R., Morel L.J., França Sde C., Zingaretti S.M., da Silva L.M., Pereira A.M., Marins M., Fachin A.L. (2010). Serotonin receptor (5-HT 2A) and catechol-O-methyltransferase (COMT) gene polymorphisms: Triggers of fibromyalgia?. Rev. Bras Reumatol..

[37] Norbury T.A., MacGregor A.J., Urwin J., Spector T.D., McMahon S.B. (2007). Heritability of responses to painful stimuli in women: A classical twin study. Brain.

[38] Williams F.M., Spector T.D., MacGregor A.J. (2010). Pain reporting at different body sites is explained by a single underlying genetic factor. Rheumatology.

[39] Lindstedt F., Lonsdorf T.B., Schalling M., Kosek E., Ingvar M. (2011). Perception of thermal pain and the thermal grill illusion is associated with polymorphisms in the serotonin transporter gene. PLoS ONE.

[40] Fillingim R.B., King C.D., Ribeiro-Dasilva M.C., Rahim-Williams B., Riley J.L. (2009). 3rd; Sex, gender, and pain: A review of recent clinical and experimental findings. J. Pain.

[41] Kato F., Abe T., Kanbara K., Ban I., Kiba T., Kawashima S., Saka Y., Mizuno Y., Fukunaga M. (2017). Pain threshold reflects psychological traits in patients with chronic pain: A cross-sectional study. Biopsychosoc. Med..

[42] Zhang Y., Ahmed S., Vo T., St Hilaire K., Houghton M., Cohen A.S., Mao J., Chen L. (2015). Increased pain sensitivity in chronic pain subjects on opioid therapy: A cross-sectional study using quantitative sensory testing. Pain Med..

